# *QuickStats:* Percentage* of Adults Aged ≥18 Years Who Had an Influenza Vaccination^†^ in the Past 12 Months, by Diagnosed Diabetes Status^§^ and Age Group — National Health Interview Survey,^¶^ 2017

**DOI:** 10.15585/mmwr.mm6749a6

**Published:** 2018-12-14

**Figure:**
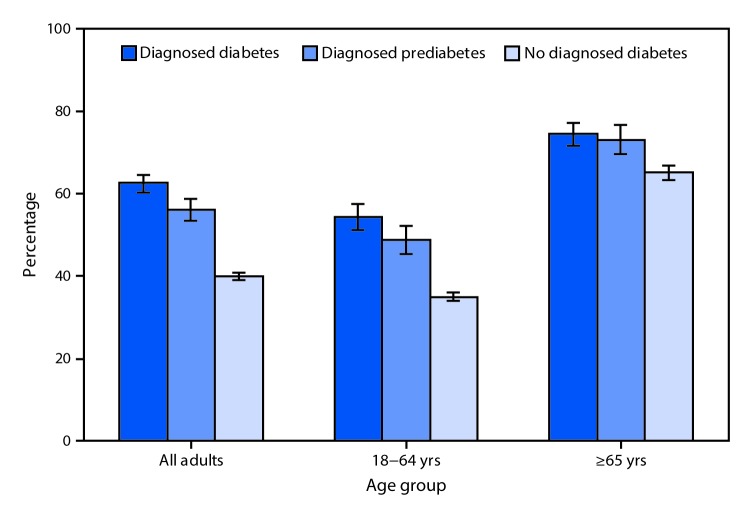
In 2017, among adults aged ≥18 years, those with a diagnosis of diabetes were more likely to have had an influenza vaccination in the past 12 months than those with a diagnosis of prediabetes (62.5% versus 56.1%); those with no diagnosed diabetes were the least likely to have had an influenza vaccination (40.1%). Among adults aged ≥65 years, influenza vaccination was higher for those with a diagnosis of diabetes (74.5%) or prediabetes (73.0%) than for those with no diagnosed diabetes (65.1%). For adults aged 18–64 years, influenza vaccination rates also were highest for those with diagnosed diabetes (54.3%), followed by those with diagnosed prediabetes (48.7%), and were lowest for those with no diagnosed diabetes (35.0%). Regardless of diabetes status, influenza vaccination rates were higher among those aged ≥65 years than among those aged 18–64 years.

**Source:** National Health Interview Survey, 2017. https://www.cdc.gov/nchs/nhis.htm.

